# Obsessive–compulsive symptoms in *ACTG1*-associated Baraitser-Winter cerebrofrontofacial syndrome

**DOI:** 10.1007/s00702-022-02544-y

**Published:** 2022-10-07

**Authors:** Theresa Göbel, Lea Berninger, Andrea Schlump, Bernd Feige, Kimon Runge, Kathrin Nickel, Miriam A. Schiele, Ludger Tebartz van Elst, Alrun Hotz, Svenja Alter, Katharina Domschke, Andreas Tzschach, Dominique Endres

**Affiliations:** 1 Department of Psychiatry and Psychotherapy, Medical Center - University of Freiburg, Faculty of Medicine, University of Freiburg, Freiburg, Germany; 2 Institute of Human Genetics, Medical Center - University of Freiburg, Faculty of Medicine, University of Freiburg, Freiburg, Germany; 3 Center for Basics in Neuromodulation, Medical Center - University of Freiburg, Faculty of Medicine, University of Freiburg, Freiburg, Germany

**Keywords:** Obsessive–compulsive disorder, OCD, Obsessive–compulsive symptoms, Baraitser-Winter cerebrofrontofacial syndrome, ACTG1

## Abstract

Symptoms of obsessive–compulsive disorder (OCD) may rarely occur in the context of genetic syndromes. So far, an association between obsessive–compulsive symptoms (OCS) and *ACTG1*-associated Baraitser-Winter cerebrofrontofacial syndrome has not been described as yet. A thoroughly phenotyped patient with OCS and *ACTG1*-associated Baraitser-Winter cerebrofrontofacial syndrome is presented. The 25-year-old male patient was admitted to in-patient psychiatric care due to OCD. A whole-exome sequencing analysis was initiated as the patient also showed an autistic personality structure, below average intelligence measures, craniofacial dysmorphia signs, sensorineural hearing loss, and sinus cavernoma as well as subtle cardiac and ophthalmological alterations. The diagnosis of Baraitser-Winter cerebrofrontofacial syndrome type 2 was confirmed by the detection of a heterozygous likely pathogenic variant in the *ACTG1* gene [c.1003C > T; p.(Arg335Cys), ACMG class 4]. The automated analysis of magnetic resonance imaging (MRI) revealed changes in the orbitofrontal, parietal, and occipital cortex of both sides and in the right mesiotemporal cortex. Electroencephalography (EEG) revealed intermittent rhythmic delta activity in the occipital and right temporal areas. Right mesiotemporal MRI and EEG alterations could be caused by a small brain parenchymal defect with hemosiderin deposits after a cavernomectomy. This paradigmatic case provides evidence of syndromic OCS in *ACTG1*-associated Baraitser-Winter cerebrofrontofacial syndrome. The MRI findings are compatible with a dysfunction of the cortico-striato-thalamo-cortical loops involved in OCD. If a common pathophysiology is confirmed in future studies, corresponding patients with Baraitser-Winter cerebrofrontofacial syndrome type 2 should be screened for OCS. The association may also contribute to a better understanding of OCD pathophysiology.

## Introduction

Obsessive–compulsive disorder (OCD) is a common mental disorder with a 12-month prevalence up to 3.6% characterized by the presence of obsessive thoughts and compulsive actions (Jacobi et al. 2014; Stein et al. 2019). Primary forms are explained by the bio-psychosocial model, including genetic, environmental, and psychoreactive factors (Stein et al. 2019). Rare secondary forms of OCD may occur in the context of genetic syndromes, such as Turner syndrome (Moonga et al. 2017). To date, little is known about syndromic OCD (Mahjani et al. 2021). The rationale of the current paper is to describe the first patient with obsessive–compulsive symptoms (OCS) and *ACTG1*-associated Baraitser-Winter cerebrofrontofacial syndrome.

Baraitser-Winter cerebrofrontofacial syndrome is a genetic disorder characterized by distinct craniofacial features, such as short stature, hypertelorism, ptosis, and a wide face (Rivière et al. 2012). Other typical characteristics include sensorineural deafness, iris and retinal coloboma, growth retardation, and intellectual disability (Dawidziuk et al. 2022). Patients also present with a brain malformation consisting of pachygyria and anterior predominant lissencephaly (Rivière et al. 2012). Other malformations, such as agenesis of the corpus callosum, cleft lip and palate, duplicated hallux, congenital heart defects, or renal tract anomalies, are observed in some cases (Di Donato et al. 2016). The cause of the syndrome are heterozygous missense mutations in one of the two ubiquitous cytoplasmic actin-encoding genes *ACTB* and *ACTG1* that encode β- and γ-actins, respectively (Verloes et al. 2015). Recent reports describe significantly milder facial phenotypes in patients with *ACTG1* mutations compared to patients with *ACTB* mutations, whereas malformations of cortical development have been common in patients with *ACTG1* mutations. Other congenital anomalies mentioned above have been associated with *ACTB* mutations (Di Donato et al. 2016).

## Methods

The presented patient received a broad diagnostic work-up and has given his signed written informed consent for this case study. The genetic analysis included a conventional chromosome analysis and array comparative genomic hybridization. For molecular genetic analysis, whole-exome sequencing was performed on DNA extracted from peripheral blood. Target enrichment of all coding genomic regions (exome) was performed with a Twist Human Core Exome Kit (Twist Bioscience) and subsequent sequencing on an Illumina platform (NextSeq1000 System, San Diego, United States) with 150-bp paired-end reads. Candidate causative variants were validated by Sanger sequencing.

The clinical diagnostic work-up included psychometric/neuropsychological testing (Obsessive–Compulsive Inventory-Revised version [OCI-R], Structured Clinical Interview for DSM-IV [SCID-I], Wechsler Adult Intelligence Scale—Fourth Edition, Autism Diagnostic Observation Schedule, Movie for the Assessment of Social Cognition, and Gnosis Facialis Testing), magnetic resonance imaging (MRI) of the brain, and electroencephalography (EEG). A combined volume- and region-based analysis method using the Magnetization-prepared rapid gradient-echo (MPRAGE) MRI sequences (https://www.veobrain.com/?page=veomorph) and an automated independent component analysis (ICA) of the EEG were also conducted (cf. Endres et al. 2017). In addition, different blood analyses (differential blood count, urinalysis, immunological screening etc.), electrocardiography (ECG), long-term ECG, transthoracic echocardiography (TTE), abdominal sonography, and neuroophthalmological examinations were conducted.

## Results

The 25-year-old male patient with a history of OCD since the age of 15 presented with a severe exacerbation of his OCS over the past year. He showed predominant washing (i.e., washing his hands about 40–50 times per day) and cleaning compulsions (i.e., ritualized room cleaning for about 4–5 h per day) fulfilling clinical DSM-5 criteria for OCD at inpatient admission. The patient could not handle everyday life activities any longer, and was therefore admitted as an inpatient. The formal diagnosis of OCD was confirmed using the SCID-IV, OCI-R (for the acquisition of OCS), resulted in a score of 40 (84% of OCD patients reach a score of ≥ 18; range 0–72).The psychiatric history examination revealed an autistic personality structure since early childhood, including distinct routines, sensitivity to noise, unusual mood reactions, and difficulties in performing in social groups. Mild developmental delays had been reported (first words between 16 and 18 months, learning to walk at 20 months). At the age of 6 years, bilateral sensorineural deafness was diagnosed. In his youth, he underwent eye surgery due to strabismus, hypermetropia, and diplopia. In the first decade of life, the patient received early intervention as well as occupational and speech therapy. At the age of 23 years, the patient underwent brain surgery after having an epileptic seizure due to intracranial cavernous bleeding. The physical examination showed an atypical phenotype of craniofacial features including bilateral ptosis, a wide face, long palpebral fissures, everted lower lids, a wide mouth, and low set ears.

Chromosome analyses were normal. Whole-exome sequencing revealed the heterozygous variant NM_001614: c.1003C > T; p.(Arg335Cys) in the *ACTG1* gene. This variant has not been reported in the population database gnomAD (the Genome Aggregation Database v2.1.1; http://gnomad.broadinstitute.org/). Several in silico algorithms (PolyPhen-2, SIFT, MutationTaster) predict a deleterious effect. According to the ACMG guidelines (Richards et al. 2015) this variant is classified as likely pathogenic (class 4). No family members were available for molecular genetic analyses. The family history was negative for OCD, autism, sensorineural deafness, and other developmental delays. The 2-year-old healthy daughter of the index patient was said to have similar facial features, but there was no detailed information available.

Neuropsychological and psychometric testing revealed a subsyndromal autistic personality and an estimated intelligence quotient (IQ) of 85. The MRI showed a small right temporal lesion after a cavernectomy. MRI analysis identified grey matter volume loss in the parietal cortex. Atrophic changes with increased cerebrospinal fluid (CSF) volume were identified in the left orbitofrontal cortex (OFC), occipital cortex of both sides, and the right mesiotemporal cortex. ICA of the EEG showed intermittent rhythmic delta activity (IRDAs) in the occipital and right temporal areas, which was increased under hyperventilation.

Laboratory tests were without relevant pathological findings. The ECG showed a QRS duration of 120 ms, RSR’ pattern in V1 and V2, and signs of left ventricular hypertrophy. The long-term ECG revealed a normal sinus rhythm with episodes of tachycardia, most likely due to sinus tachycardia. The TTE detected a normal sized heart with normal systolic and diastolic functions. An aortic bulb dilatation of 40 mm was detected. The abdominal sonography showed no pathology of the kidneys but revealed two small gallbladder polyps. The neuroophthalmological examination revealed abnormal papilla on both sides with temporal pallor and a nasal displacement of the retinal vessels. No concrete signs for coloboma were detected.

**Fig. 1:**
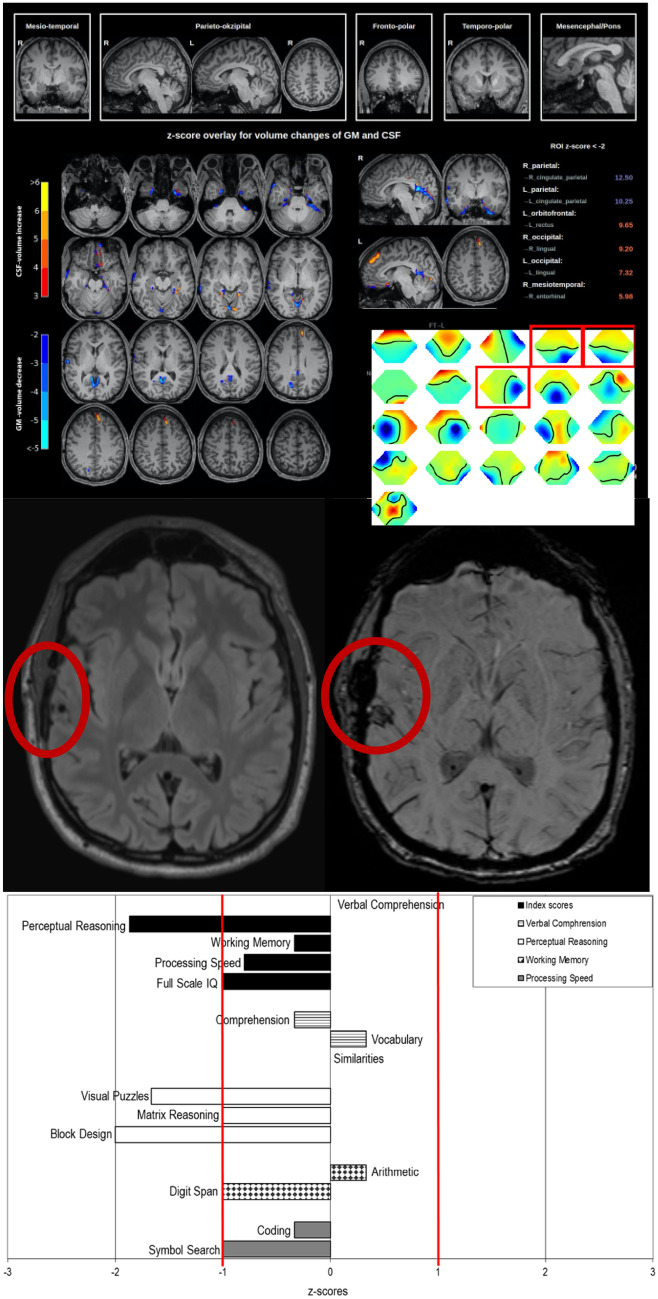
A combined volume- and region-based analysis method (above) using the MPRAGE magnetic resonance imaging (MRI) sequences identified grey matter (GM) volume loss in the right (z-score: 12.5) and left (z-score: 10.25) parietal cortex. Atrophic changes with increased cerebrospinal fluid (CSF) volume were identified in the left orbitofrontal cortex (z-score: 9.65), right (z-score: 9.2) and left (z-score: 7.32) occipital cortex, and the right mesiotemporal cortex (z-score: 5.98). FLAIR and SWI sequences (central) showed a small brain parenchymal defect right temporal with hemosiderin deposits after cavernomectomy. The inset shows the electroencephalography independent component analysis maps. The components showing intermittent rhythmic delta activity (right and left occipital, right temporal) are framed. The Wechsler Adult Intelligence Scale—Fourth Edition yielded an overall intelligence quotient of 85 (processing speed score of 88, working memory score of 95, perceptual/logical reasoning score of 72, and verbal comprehension score of 100; bottom). *FLAIR* fluid attenuated inversion recovery, *MPRAGE* magnetization prepared rapid gradient echo, *NPH* normal pressure hydrocephalus, *SWI* susceptibility-weighted imaging

Due to a seizure in the past, the patient had been treated temporarily with levetiracetam (max. 2.000 mg per day). For symptoms of OCS, sertraline (uptitrated to 200 mg per day) was given. This treatment resulted in reduction of washing and cleaning compulsions. During the course of disease, the patient discontinued sertraline, and a switch from levetiracetam to lamotrigine was initiated. In addition, the patient improved under cognitive behavioral psychotherapy with exposure and response prevention (Fig. 1).

## Discussion

A patient with OCS and Baraitser-Winter cerebrofrontofacial syndrome type 2 is presented. The patient fulfilled the clinical criteria of OCD, in addition he suffered from autistic personality structure, below average IQ, craniofacial dysmorphic features, sensorineural hearing loss, and sinus cavernoma as well as subtle cardiac and ophthalmological involvement. The diagnosis of Baraitser-Winter cerebrofrontofacial syndrome type 2 was confirmed by the detection of a heterozygous likely pathogenic variant in the *ACTG1* gene [c.1003C > T; p.(Arg335Cys)].

A systematic Medline search (search strategy: “Baraitser-Winter AND (OCD OR obsessive–compulsive disorder OR OCS OR obsessive–compulsive symptoms)”; retrieved 30 July 2022) revealed no comparable cases, rendering this the first report of Baraitser-Winter cerebrofrontofacial syndrome with OCS.

At the pathophysiological level, a common pathophysiology of the Baraitser-Winter cerebrofrontofacial syndrome type 2 and OCS would be possible. Baraitser-Winter cerebrofrontofacial syndrome in our patient was caused by a likely pathogenic variant in the cytoplasmic actin-encoding gene *ACTG1* (Rivière et al. 2012). Epigenetic studies investigating DNA methylation in patients with OCD suggested differentially methylated genes concerning the regulation of actin cytoskeleton (KEGG hsa04810), cell adhesion molecules (CAMs, KEGG hsa04514), and actin binding (GO:0003779) to be associated with OCD (Yue et al. 2016). Therefore, OCD might be associated with impaired regulation of actin cytoskeleton and actin binding (Yue et al. 2016). MRI findings in the presented patient also suggested brain involvement of Baraitser-Winter cerebrofrontofacial syndrome. The atrophic changes in the OFC/parietal cortex on both sides and the right mesiotemporal cortex would be compatible with the cortico-striato-thalamo-cortical network dysfunction well established in OCD (Stein et al. 2019). From the OFC originates the ventral motivational loop, which is thought to be responsible for stimulus-based motivational behavior, frontoparietal networks which regulate cognitive control, and frontolimbic loops (via amygdala) which are responsible for fear extinction (Stein et al. 2019).

As a limitation, it should be mentioned that a “psychoreactive” development of primary OCD in the context of everyday difficulties caused by characteristics along with a genetic syndrome, such as hearing loss and lower cognitive performance, cannot be excluded. Nevertheless, the diagnosis contributed to the reduction of self-stigma in the patient. The patient improved with guideline-based therapy.

In summary, this case provides evidence of OCS in *ACTG1*-associated Baraitser-Winter cerebrofrontofacial syndrome. More patients with Baraitser-Winter cerebrofrontofacial syndrome should be screened for OCS. In this context, it should also be studied whether washing and cleaning compulsions—that predominated in the patient described here—in particular can be identified. If the association is confirmed, this could not only improve the clinical management of patients with Baraitser-Winter cerebrofrontofacial syndrome but could also provide pathophysiological insights into OCD.

## Data Availability

All necessary data can be found in the paper.
